# Development of a Home Meal Replacement Product Containing Braised Mackerel (*Scomber japonicus*) with Radish (*Raphanus sativus*)

**DOI:** 10.3390/foods10051135

**Published:** 2021-05-19

**Authors:** Gabriel Tirtawijaya, Seung Rok Kim, Woo Hee Cho, Jae Hak Sohn, Jin-Soo Kim, Jae-Suk Choi

**Affiliations:** 1 Seafood Research Center, IACF, Silla University, 606, Advanced Seafood Processing Complex, Wonyang-ro, Amnam-dong, Seo-gu, Busan 49277, Korea; tirtawijayag@yahoo.com (G.T.); tmdfhr88@naver.com (S.R.K.); ftrnd3@silla.ac.kr (W.H.C.); jhsohn@silla.ac.kr (J.H.S.); 2 Department of Food Biotechnology, College of Medical and Life Sciences, Silla University, 140, Baegyang-daero 700 beon-gil, Sasang-gu, Busan 46958, Korea; 3 Department of Seafood and Aquaculture Science, Gyeongsang National University, 38 Cheongdaegukchi-gil, Tongyeong-si, Gyeongsangnam-do 53064, Korea

**Keywords:** high-frequency defroster, HMR, mackerel, superheated steam roaster, quick freezing

## Abstract

The coronavirus disease pandemic has contributed to increasing convenience in food preferences. Home meal replacement (HMR) products are ready-to-eat, -cook, or -heat foods, providing convenience for consumers. We developed a HMR product containing mackerel as a protein- and lipid-rich source using various food-processing technologies to maintain its nutritional content and prolong shelf life. The HMR product contained mackerel, radish, and sauce in a ratio of 5:1:4. Raw frozen mackerels were thawed by using a high-frequency defroster before being braised using a superheated steam roaster. Response surface methodology was employed to obtain the optimal heating conditions of 181 °C for 9 min. The final test HMR product was packed in a polypropylene plastic bowl prior to freezing at −35 °C for 1 h using a quick freezing system. The HMR product developed using these technologies exhibited stable microbiological and chemical properties for 90 days of storage. Sensory scores gradually decreased with increasing storage temperature and time. Protein content in the HMR product was 13%, 40% of which comprised essential amino acids; lipid content was 13.4%, 18% of which was composed of docosahexaenoic acid. The HMR product can preserve its quality and is considered safe for consumption for up to 40 months of storage at −18 °C.

## 1. Introduction

The coronavirus disease (COVID-19) emerged at the end of 2019 and was declared a pandemic in March 2020 by the World Health Organization. Since then, lifestyles have changed following social distancing restrictions and mandatory lockdowns in some areas. COVID-19 has increased the consumption pattern of ready-to-eat and ready-to-cook foods. During the lockdown period, interest in the search for delivery services and recipes was higher than that for restaurant food [1]. Preferences for convenience food and long shelf-life products, as well as products for home meal preparation, such as flour and pastry ingredients, have increased [2].

Food innovation in response to ongoing global pandemics is a challenge. The consumption rate of convenience and simple foods has also grown by 15.3% annually [3]. Innovations in the convenience of food varieties are needed to provide consumers with a wide range of alternatives. Home meal replacement (HMR) products are convenient foods that are ready to eat, ready to cook, ready to prepare, and ready to heat. HMR products are designed to meet daily dietary needs with suitable quality and optimal nutrition and prolong food shelf life through innovative preparation and heating technologies. 

Fish is a widely accepted food source and is suitable for use as a raw material in HMR production because of its nutritional value. One such widely consumed fish is mackerel (*Scomber japonicus*). Over one million tons of mackerel are produced annually in Korea, China, and Japan [4]. Mackerel is known to contain compounds beneficial for brain development, such as eicosapentaenoic acid (EPA, 2–8% of total fatty acids), docosahexaenoic acid (DHA, 13–23.4% of total fatty acids), proteins (18.4–20.4%), and lipids (9.0–23.2%) [5,6]. Mackerel, which provides excellent nutrition and is abundantly available, should be utilized as a sustainable resource for public health, and one of its applications could be in the development of HMR products. The development of mackerel-based HMR products has the potential to broaden the distribution of mackerel consumption while also extending its shelf life.

Mackerel can be cooked using various methods, including frying, boiling, smoking, and braising. Braised mackerel with radish is a popular dish in Korean cuisine and is known as *godengeo-mu-jorim* [7]. Braised mackerel is prepared by combining a sauce with fish and radish to improve its taste and eliminate the fishy smell. A HMR product containing braised mackerel with radish might become a popular healthy convenience food. Several technologies can be applied to maintain the quality of a HMR product containing braised mackerel with radish. Several technologies applied in the heating of HMR products, such as those containing hagfish [8], Pacific saury [9], squid [10], and pen shells [11], have been reported. In the aforementioned studies, HMR products were prepared using a superheated steam roaster (SSR), which can maintain food texture, improve product quality, reduce oxidation, and shorten the heating process. To date, these findings have primarily focused on heating methods, although material preparation and postproduction freezing are also required to produce high-quality products.

Frozen materials are commonly used in industrial food manufacturing, including fish, to maintain sustainability throughout the season. It requires proper treatment with a thawing process to produce quality products. High-frequency defrosters (HFDs) have been used to thaw meat and seafood products. A HFD operates at a frequency of 27.12 MHz and facilitates a shorter thawing time and less deterioration of product quality, resulting in reduced drip loss [12]. 

Generally, HMR products are frozen to expand their distribution and extend their shelf life. It is critical to evaluate the freezing methods to ensure that the products have similar properties when they reach consumers. The freezing rate influences the quality of frozen food; for example, quick-freeze salmon contains smaller ice crystals, resulting in less damage to the fish muscle [13]. Ice crystals melt and disengage from the muscle during the thawing process, along with proteinase and pro-oxidants, ultimately reducing muscle quality [14].

Research on the development of braised mackerel with radish as a HMR product has never been conducted before. In this study, newly developed HMR braised mackerel with radish involved a series of processes to maintain product quality and ensure the product is acceptable to consumers as a home meal. HMR braised mackerel with radish is classified as a ready-to-heat food, so that consumers can easily reheat the product using a microwave before serving. We evaluated the development of a HMR product containing braised mackerel with radish using each of the above-mentioned processes to ensure that consumers receive a product with optimal sensory quality and nutritional content. These processes involved HFD for thawing frozen fish, SSR for heating, and a quick freezing system to freeze the final products for storage. Response surface methodology (RSM) was used to obtain the optimal heating conditions using SSR. RSM is a cost-effective method for assessing the interaction of independent variables using a small number of experiments [15]. The evaluation process emphasizes the sensory properties of the product, which is complemented by physicochemical and microbiological conditions. The effects of processing technologies on a HMR product containing braised mackerel with radish were also evaluated to estimate the shelf life.

## 2. Materials and Methods

### 2.1. Preparation of the Raw Materials

Mackerel (*S. japonicus*) samples were obtained from EBADA Fishery Co. Ltd., Busan, Korea, under frozen conditions. Fresh radish samples (*Raphanus sativus*) were purchased from a local market in Busan, Korea. Frozen mackerel samples were placed in zip-lock plastic bags and thawed using three different methods: air thawing (AT, 18–20 °C), water thawing (WT, 22–24 °C), and high-frequency defroster (HFD; TEMPERTRON FRT-10, Yamamoto Vinita Co. Ltd., Osaka, Japan). The temperature gap between the HFD (internal) and room temperature was within 3 °C. The weights of mackerel samples before and after the thawing process were measured to evaluate their drip loss percentage.

### 2.2. Braised Mackerel with Radish Processing

Thawed mackerel samples were steak-sliced into a size of 1.5 × 4.5 × 6.0 cm (thickness × width × length). The mackerel slices were immersed in 2% saltwater for 5 min to reduce the fishy odor. Fresh radish samples were washed using running tap water, peeled off, and cut into a size of 0.5 × 2.0 × 2.0 cm (thickness × width × length).

A braised mackerel with radish HMR product sample was prepared in a single stainless bowl (Ø, ±13.0 cm; volume, ±550 mL) by mixing mackerel, radish, and sauce in a ratio of 5:1:4. The ingredients of the sauce are listed in Table 1. A total of 200 g of braised mackerel with radish was cooked using an SSR at different temperatures and times based on the design of the experiment using RSM. After roasting, the samples were cooled and moved to a polypropylene plastic bowl (Ø, 10.0 cm; volume, 150 mL; New Ecopack Co., Ltd., Jeonju, Korea) prior to sealing with a TPS-TS3T packaging machine (TPS Co., Ltd., Gyeonggi-do, Korea) at 180 °C for 5 s.

**Figure 1:**
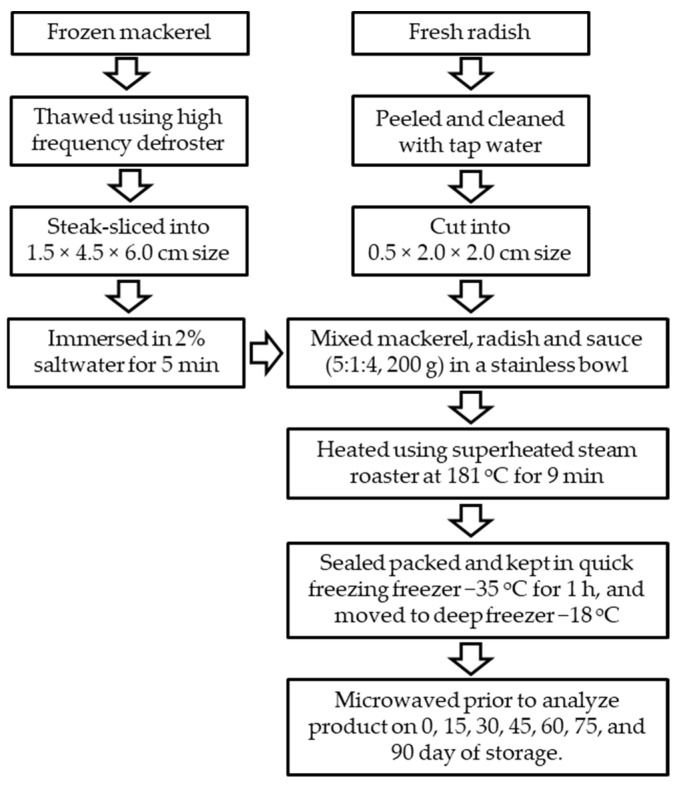
The processing scheme of home meal replacement braised mackerel with radish.

To optimize the initial storage conditions, packaged samples were stored in a freezer using two different methods: slow freezing and quick freezing. Quick freezing treatment was performed in a quick freezer QF-700 (Alpha Tech Co., Ltd., Incheon, Korea) at −35 °C for 1 h, while slow freezing treatment was performed in a regular freezer (Samsung CRF-114 CD, Samsung Electronics Co., Ltd., Seoul, Korea) at −24 °C for 3 h. After freezing at the indicated time, all the samples were stored in a deep freezer DF35035 (IlShin BioBase Co., Ltd., Dongducheon, Korea) at three different temperatures of −13, −18, and −23 °C for evaluating the shelf life. The processing scheme of the braised mackerel and radish is shown in Figure 1.

### 2.3. Optimization of Heating Conditions

RSM was used to optimize the heating conditions. The heating conditions were optimized by using two factors as independent variables (temperature and time) with a five-level central composite design. The design generated 11 runs consisting of low, central, and high factor levels. The central point was selected based on a preliminary study in which the temperature varied from 150–200 °C. Braised mackerel with radish prepared at 150 and 200 °C for 10 min had poor quality (undercooked and overcooked, respectively), while the dish prepared at 180 °C had good quality. Thus, 180 °C and 10 min were chosen as the center points. The low and high points were calculated using the RSM design. The coded values −1.414, −1, 0, 1, and 1.414, as well as their actual values of independent variables, are shown in Table 2. 

The optimization was evaluated by measuring the overall acceptability (sensory analysis) of the final product and the hardness of the radish. The optimization target value for overall acceptability was 9 (as the maximum score in the sensory analysis), while hardness was 724 g, based on a previous study by Endo et al. [16]. The effect of two factors (Y) was modeled using a polynomial response surface with the prediction values using the following equation:

Y = b_0_ + b_1_X_1_ + b_2_X_2_ + b_11_X_1_^2^ + b_22_X_2_^2^ + b_12_X_1_X_2_, (1)

where b_0_, b_1_, b_2_, b_11_, b_22_, and b_12_ are the constant regression coefficients of the model, and X_1_ and X_2_ are the independent variables. 

### 2.4. Sensory Analysis

The sensory properties, such as color, aroma, flavor, texture, and overall acceptability, were evaluated for three purposes: (1) to optimize the heating conditions in RSM design, (2) to evaluate the freezing method, and (3) to estimate the shelf life of the product. The products were analyzed by 21 trained panelists (age range, 25–40 years). The braised mackerel was heated in a microwave (RE-M50, Samsung Electronics Co. Ltd., Seoul, Korea) for 2 min (700 W) before analysis. The sensory properties were scored by the panelists using a hedonic scale of 1–9, representing extreme dislike to extreme like. Sensory properties with a score below the threshold (score 5) were categorized as unacceptable [17]. Sensory analysis was performed with the approval of the Silla University Institutional Review Board (Busan, Korea). 

### 2.5. Physicochemical Analysis

The physicochemical properties, including hardness, pH, thiobarbituric acid reactive species (TBARS) levels, and volatile basic nitrogen (VBN) values, were measured using the methods described in a previous study [10]. The pH, TBARS, and VBN values were measured in the final product after quick freezing and storage at −18 °C. These parameters were measured on days 0, 15, 30, 45, 60, 75, and 90 and were preceded by heating the products in the microwave, as mentioned in the sensory analysis. Radish hardness was evaluated to determine the optimal heating conditions. Hardness values were measured using a texture analyzer (Brookfield, Middleboro, MA, USA). 

### 2.6. Microbial Analysis

The microbial composition of the product was evaluated based on the total bacterial count (TBC) and proportion of *E. coli*, *Staphylococcus aureus*, and *Salmonella*. This evaluation was performed according to the methods described in a previous study [8]. Microbial analysis was conducted on days 0, 15, 30, 45, 60, 75, and 90 at three different storage temperatures of −13, −18, and −23 °C. The microorganism count data are expressed as log CFU/g.

### 2.7. Nutritional Quality Analysis

The nutritional quality of braised mackerel with radish, such as the amounts of calories, carbohydrates, fats, saturated and trans-fat, cholesterol, protein, sugar, sodium, moisture, and ash were analyzed according to the method of the Association of Official Analytical Chemists [18]. Fatty acid levels were measured quantitatively and compared to the retention time of the standard FAME mix 37 components (Supelco Inc., Bellefonte, PA, USA). A gas chromatography-mass spectrophotometer (Shimadzu GC–2010 Plus) equipped with a flame ionization detector (FID; Shimadzu Corp., Kyoto, Japan) was used for fatty acid analysis. Fatty acids were separated using a Supelco column SP–2560 (100 m × 0.25 × 0.25 µm; Supelco Inc.) with nitrogen gas as a carrier at 0.94 mL min^−1^ flow rate and 1:50 split ratio. An oven temperature of 240 °C was obtained by a gradual increase of 3.5 °C min^−1^ from 100–200 °C in the fatty acid separation system.

Amino acid composition was analyzed using a Hitachi L-8900 Amino Acid Analyzer (Hitachi High-Tech Corp., Tokyo, Japan). The samples were hydrolyzed by 6N HCl at 110 °C for 24 h. HCl present in the hydrolysates was removed by adding distilled water and performing drying treatments twice. Hydrolysates were prepared for injection by diluting with 0.02 N HCl and filtered using a membrane filter. The amino acid compositions of the samples were compared to those of the 18 components of the amino acid standard. 

### 2.8. Shelf Life Analysis

The shelf life of the HMR product containing braised mackerel with radish was analyzed according to the guidelines of the Ministry of Food and Drug Safety (MFDS), Korea. The data related to overall acceptability and TBC were analyzed using the Visual Shelf Life Simulator for Foods program by MFDS (https://www.foodsafetykorea.go.kr (accessed on 15 February 2021)) based on the Arrhenius kinetic approach for estimating the shelf life of the product. Three different storage temperatures of −13, −18, and −23 °C for 90 days were used to obtain the constant rate for the Arrhenius method. The changes in product quality are usually modeled by means of a zero- or first-order reaction [19]. The reaction order with the largest coefficient of determination (*R*^2^) was selected to determine the Arrhenius equation in that order. Determination of the Arrhenius equation was obtained by plotting ln k(reaction rate constant), and 1/*T* (temperature in Kelvin scale). The Arrhenius equation is shown as follows:

(2) $$k=A_{0}e^{\frac{E_{a}}{RT}}$$

where $A_{0}$ is the Arrhenius constant, $E_{a}$ is the activation energy (J/mol), *R* is the universal gas constant (8.31 J/mol K), and *T* is the absolute temperature (K). The critical point or rejection criterion for overall acceptance of sensory evaluation was 5, and for TBC it was 5 log CFU/g. The calculated shelf life was multiplied by a safety factor of 0.8, to better predict the shelf life [20].

### 2.9. Statistical Analysis

Data for the optimization of heating conditions were analyzed using the RSM. Analysis of variance (ANOVA) was used for quadratic polynomial and lack of fit determination in the heating condition optimization experiment. The data on drip loss, sensory properties in freezing methods, and chemical properties were analyzed by ANOVA followed by Tukey’s multiple comparison tests at a significance level of *p* < 0.05. The data on the effect of storage time and temperature on the prepared HMR product were analyzed using a two-factorial design followed by Tukey’s multiple comparison tests for the interaction at a significance level of *p* < 0.05. All statistical analyses and surface plots were performed using Minitab ver. 19.0 (Minitab LLC, State College, PA, USA).

## 3. Results and Discussion

### 3.1. Drip Loss of Frozen Raw Mackerel

**Figure 2:**
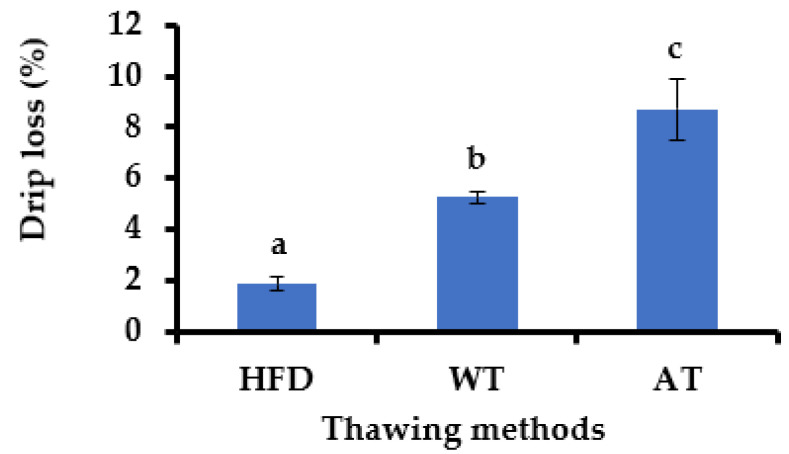
Drip loss (%) of frozen raw mackerel after thawing process using high-frequency defroster (HFD), water thawing (WT), and air thawing (AT). The data are shown as the mean ± standard error of the mean (SEM). The means with different letters are significantly different between thawing methods (Tukey’s test, *n* = 3, *p* < 0.05).

In this study, frozen mackerel samples were used to prepare a HMR product. To maintain the quality of the product, we evaluated the drip loss of thawed mackerel using three different methods, as shown in Figure 2. The thawing method significantly affected the drip loss of the frozen mackerel. HFD reduced drip loss significantly compared to WT and AT methods (*p* < 0.05).

Conventional thawing methods with WT and AT required a longer time (60 and 90 min, respectively) than the HFD method (20 min) to achieve a defrost state. A short thawing time maintains fish quality and minimizes mechanical damage to cell membranes [21,22]. Drip loss in frozen mackerels is caused by the release of free water from the muscles. Water-soluble proteins leach out during thawing periods, lowering the quality of the product [23]. Freezing-thawing processes may damage cells, lead to the denaturation of protein, and dehydrate muscles [24]. Farag et al. [25] showed that the high-frequency thawing method was more efficient in reducing drip and micronutrient losses than conventional thawing. The thawing process can worsen the texture of frozen fish. Genç et al. [26] reported that thawing fish filets decreased the water holding capacity and increased hardness. Low drip loss indicates that HFD can prevent the loss of nutrients and maintain the yield rate during defrosting, even without water use. Thus, the HFD method for defrosting frozen mackerel was selected for use in the further steps of this study.

### 3.2. Optimal Heating Conditions

Two variables, overall acceptance and hardness, were selected to evaluate the optimal heating conditions using an SSR. The overall acceptance score represented the sensory properties of braised mackerel with radish, while hardness values represented radish conditions. The hardness value was measured only on radishes because the range of temperature and time in the optimization experiment was sufficient for heating the mackerel, but it varied in radishes. In this study, RSM was used to optimize the heating conditions to achieve a specific set of objectives. The output of the RSM was the optimum combination of time and temperature as independent variables and overall acceptance and hardness as dependent variables.

Table 3 shows the predictive models that reflect the relationship between overall acceptance or hardness and the heating conditions. These models can be used to estimate the score of overall acceptance and hardness of braised mackerel with radish when cooked at different times and temperatures at SSR. The predicted and actual values from the regression model and variable measurements are shown in Table 4. The *R*^2^ and lack of fit values were analyzed to evaluate the adequacy of the developed models and their predictive values. The results showed that the models were significant at a 95% confidence level (*p* < 0.05). The *R*^2^ values of both predictive models were close to 100% (i.e., perfect fit), indicating that the models explained 98.82 and 96.93% of the variability of the response data around its mean. The *p*-values of lack of fit of both models were not significant (*p* > 0.05), indicating that the models were fit and adequate for predicting the heating conditions of braised mackerel with radish.

**Figure 3:**
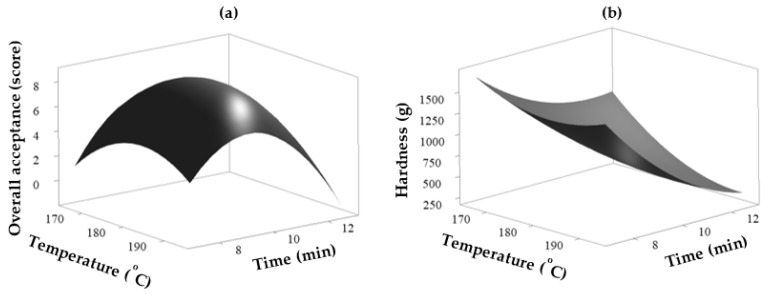
Response surface graph developed from RSM for (**a**) overall acceptance (score) and (**b**) hardness (g) of braised mackerel with radish at the designated superheated steam roaster temperature (°C) and time (min).

The three-dimensional response surface graph of overall acceptance showed an increased score with increasing temperature and time but decreased when it reached the optimal condition of 180.625 °C for 9.0316 min (Figure 3a). The hardness value of the radish decreased continuously with an increase in the heating time and temperature (Figure 3b).

At temperatures of over 180 °C for more than 10 min, the braised mackerel with radish sauce was scorched and had a bitter taste. The radish texture also became mushy because of overcooking. Conversely, at lower temperatures and shorter heating times, the radish texture was hard and uncooked. The optimal heating conditions of braised mackerel with radish using SSR were 180.625 °C for 9.0316 min (Table 5). The predicted values of response for overall acceptance and hardness were 8.49 and 740.74 g, respectively. The actual experimental values obtained from the temperature at 181 °C for 9 min were 8.55 ± 0.10 and 761.83 ± 78.33 g for overall acceptance and hardness, respectively. The differences in the recommended heating conditions were measured using RSM because the SSR setting option was not available. Therefore, the recommended temperature and time values were rounded off. The HMR product containing braised mackerel with radish was cooked under these optimal conditions and analyzed in subsequent experiments.

The utilization of SSR in this study provides a faster process for the production of braised mackerel with radish. In a single running process, mackerels were exposed to both roasting and stewing treatments. The heating time was associated with the moisture and hardness of the product. A longer heating process results in increased hardness owing to less moisture [27]. Under optimal heating conditions, the product is neither overcooked nor undercooked. Heating mackerel under SSR provides a lower weight loss than an electric pan [28]. Several studies have shown the superiority of SSR to other heating methods in the maintenance of moisture, prevention of lipid oxidation, and reduction of the time taken for heating [8,10,29,30]. 

### 3.3. Effect of Freezing Method on the Sensory Properties of Braised Mackerel with Radish

The HMR products containing braised mackerel with radish were assessed for their quality after freezing. Freezing of HMR products is important for promoting market distribution. Freezing inhibits microorganism growth and enzymatic activity to maintain the nutritional properties of food. However, ice crystal formation may have a detrimental effect on food texture and cause membrane disruption, leading to oxidation [31]. A proper freezing method can ensure the delivery of quality products to the consumers. Improvements in the freezing phase are also linked to an increase in the freezing rate, which can be accomplished by more advanced refrigeration systems. The HMR products were frozen in two separate freezers according to their freezing rate: slow freezer and quick freezer. After freezing, all the tested HMR products were reheated using a microwave and evaluated by panelists. The sensory properties of the HMR products before and after freezing are shown in Table 6.

The HMR products were reheated using a microwave to recapitulate the experience of the product as it is used by customers. The frozen HMR products did not differ significantly in color, aroma, flavor, and overall acceptance, but the freezing treatment significantly affected their texture score (*p* < 0.05). Compared with the HMR products before freezing, the texture score slightly decreased following quick freezing (1.18%), but it significantly decreased following slow freezing (5.67%). Zhu et al. [32] reported that frozen salmon showed physical changes in texture and weight loss after thawing due to ice crystal formation. Furthermore, Ottestad et al. [33] observed that raw salmon changed color under frozen conditions but returned to their initial color after thawing.

Slow freezing has been known to result in the formation of large extracellular ice crystals, which may cause severe tissue damage in frozen foods [34]. Alizadeh et al. [22] reported that the use of a slow-freezing method for fish resulted in the development of large ice crystals and significantly destroyed the muscle fibers. Quick freezing can remove heat faster than slow freezing. The heat removal rate determines the crystal growth rate [35]. In addition, there is less disruption to the cell walls in the quick freezing system owing to the rapid rate of heat removal and ice formation [36]. Because quick freezing yields better results in terms of sensory properties, particularly in the texture score, we subjected the HMR products containing braised mackerel with radish to the quick freezing method to evaluate their shelf life throughout 90 days of storage.

### 3.4. Chemical Properties of Braised Mackerel with Radish

The HMR products containing braised mackerel with radish were prepared through a high-frequency thawing process for frozen mackerel, cooked in an SSR at optimal time and temperature, and frozen by the quick freezing system. The chemical properties, including pH, TBARS, and VBN values, were evaluated during 90 days of storage at −18 °C. A storage temperature of −18 °C was used because it is the temperature set on most freezers in the market. The pH, TBARS, and VBN values increased slightly but did not vary significantly over the storage time. These values represent the quality of the food during storage. The pH and VBN values can be used to describe the spoilage conditions of food, such as fish [37], while the TBARS value describes lipid peroxidation in food [38].

In this study, the pH values of the HMR product ranged from 5.97 ± 0.07 to 6.07 ± 0.03 within 90 days of frozen storage, comparable to the pH values of fresh fish, which varies from 5.5 to 6.6 [39]. The pH values were consistent with the findings reported by El-Dengawy et al. [40], in which frozen mackerel increased their pH values from 5.96–6.20 after 4 months of storage. Lipid oxidation of the HMR products was detected using TBARS analysis. The TBARS values of the HMR products ranged from 2.15 ± 0.11 to 2.40 ± 0.03 mg MDA/kg. The products were considered to be in a perfect condition because they had a value less than 3 mg MDA/kg [39]. Freezing treatment improved the stability of the lipids of processed mackerel and extended the shelf life. Additionally, a heating method with low oxidation possibility can help preserve the initial quality of the product [41]. 

SSR offers some value in processed foods, such as appropriate texture, and prevents oxidation during processing [29,42]. Low TBARS values indicate that the quality of HMR products is suitable, lacks rancidity, and is appetizing. In addition to lipid oxidation, high VBN levels also generate an unpleasant aroma. The amount of VBN in fish products is related to the activities of microorganisms and endogenous enzymes [43]. The VBN values of HMR products ranged from 11.67 ± 0.51 to 12.53 ± 1.12 mg% during 90 days of storage. This explains why microorganism and enzyme activities were inhibited, which might have occurred due to heating and freezing processes. Our HMR products are rated as good quality due to a VBN value of less than 25 mg% [39]. These findings show that the use of heating technology for preparing HMR product containing braised mackerel with radish can preserve the quality of the product by maintaining the rate of lipid oxidation and preventing changes in the levels of spoilage-related chemicals.

### 3.5. Nutritional Compositions of Braised Mackerel with Radish

The nutritional quality of HMR products containing braised mackerel with radish was evaluated based on proximate, fatty acid, and amino acid compositions (Table 7, Table 8 and Table 9). Table 7 shows the biochemical profile of the HMR product containing braised mackerel with radish. The results showed that moisture content (63.21%) was the highest compared to the others, followed by fat (13.42%) and protein (13.03%) contents. Moon et al. [44] reported that the moisture content of raw mackerel was 65.5% and decreased to 49.2% after it was fried in a pan. Differences in the preparation process with respect to sauce addition and the utilization of SSR, which can help retain moisture during heating, led the HMR product having a higher moisture content than previously reported. Higher moisture content also resulted in lower protein and fat contents than those reported by Moon et al. [44] (24.1 and 22.2%, respectively). Tirtawijaya et al. [8] reported that proximate content, which includes sodium, carbohydrate, protein, and fat, increased during the heating process. The addition of sauce to the HMR product as a seasoning increased the sodium and carbohydrate contents. The increase in such contents is related to an increase in calories. Based on the nutritional intake standard for Koreans, the HMR product containing braised mackerel with radish meets 10% of calories, 24% of proteins, 17% of sodium, 2.7% of carbohydrates, 4.3% of sugars, 25.8% of fat, and 8.9% of cholesterol daily intake of 2000 kcal [45].

Table 8 shows the amino acid profiles of the HMR product containing braised mackerel and radish. The total amino acid content was 12.94 g per 100 g HMR product, which was dominated (above 8%) by glutamic acid, aspartic acid, lysine, and leucine. These amino acids contribute to the taste and flavor of seafood [46]. Glutamic acid is responsible for the umami flavor, while alanine and glycine contribute to sweetness in food [47]. The total essential amino acid (EAA) content was 40%, including valine, leucine, isoleucine, methionine, phenylalanine, threonine, lysine, histidine, and tryptophan, which was lower than that of nonessential amino acids (NEAA). However, both EAAs and NEAAs play important roles in human health. Wu [48] classified arginine, cystine, leucine, methionine, tryptophan, tyrosine, aspartate, glutamic acid, glycine, proline, and taurine as functional amino acids in human nutrition. According to Mohanty et al. [49], amino acids play important roles in cell division, growth, tissue repair, and immune function. These results show that the HMR product containing braised mackerel and radish may provide amino acids to support human health.

The fatty acid profiles of the HMR product containing braised mackerel with radish are shown in Table 9. The total fatty acid content was 1.1 g per 100 g of the product, consisting of total unsaturated fatty acids (0.79 g), which was higher than that of saturated fatty acids (0.36 g). The three major fatty acids in the HMR product were palmitic acid (19.88%), oleic acid (25.65%), and DHA (18.07%). These results are in accordance with those of Celik [50], who reported that the levels of major fatty acids in mackerel captured in different seasons varied, including palmitic acid (19.36–26.59%), oleic acid (4.13–10.69%), and DHA (17.12–24.94%). Moreover, the levels of polyunsaturated fatty acids in mackerel, such as DHA and EPA, are the highest during winter. This report can be considered when selecting mackerel as a material for the development of HMR products. Various heating methods have been reported to affect the fatty acid content of mackerel. The frying pan method significantly decreased the DHA content of the mackerel, but the oven and microwave methods did not differ significantly [44]. The HMR product containing braised mackerel with radish was cooked using the SSR method, which has comparable characteristics to an oven and a microwave, but it is markedly better because it prevents the oxidation of the product [29]. Consequently, the DHA content of the HMR product can be preserved. DHA helps maintain memory and reduces cognitive impairment, and is used to treat cardiovascular diseases [51]. The high DHA content in the HMR product (2.1 g per 100 g) is beneficial for health.

### 3.6. Evaluation of Storage Effect on the Quality of Braised Mackerel with Radish

The HMR product containing braised mackerel with radish was frozen using quick freezing and stored in a deep freezer at three different temperatures of −13, −18, and −23 °C. The quality of the test HMR products was evaluated based on their sensory and microbiological properties. Sensory properties were evaluated during 90 days of storage. The data on color, aroma, flavor, texture, and overall acceptance score obtained from 21 trained panelists are shown in Table 10. HMR products at the lowest storage temperature of −23 °C maintained their color, aroma, and flavor for 90 days, but texture and overall acceptance score decreased significantly on days 60 and 90, respectively (*p* < 0.05). At a temperature of −13 °C, color and flavor scores decreased significantly on day 60, while texture and overall acceptance scores decreased significantly on day 30 (*p* < 0.05). Storage temperature did not affect the aroma scores of the HMR products. The sensory properties of foods are correlated with their physicochemical changes [52]. The presence of oxygen causes lipid oxidation during frozen storage, resulting in the loss of nutrition, color, taste, and texture. Lipid oxidation products may cause protein oxidation and alter food texture [53]. Changes in the chemical properties of the product over 90 days were not significantly different. This might be the reason why sensory properties had scores above 8 in all HMR products at all storage temperatures. 

The perception of consumers can be used to estimate the shelf life of food products. Changes in sensory properties can determine the shelf life of hygienic or microbiologically stable foods [54]. Overall, the sensory properties were excellent, with scores higher than 8 during 90 days of storage. The overall acceptance scores gradually decreased with increasing storage temperatures and times; hence, these scores were used to represent the estimated shelf life. The shelf life of the HMR products was estimated using a program simulation from MFDS based on the Arrhenius equation model. The overall acceptance was established to meet first order reaction kinetic. Table 11 shows the linear regression of the first order reaction kinetic of overall acceptance. The shelf life was estimated at a storage temperature of −18 °C. A temperature of −18 °C is required for frozen food storage [55]. The HMR product can be stored for up to 48 months at −18 °C with a target overall acceptance score threshold of 5. 

To assess the safety of the HMR products, we evaluated the microbiological conditions during 90 days of storage. Pathogenic bacteria, including the *coliform* group, *Staphylococcus aureus*, and *Salmonella* spp., were not detected in the HMR products during 90 days of storage. The absence of these pathogenic bacteria indicated that the raw materials and development processes were hygienic. The TBC (3.11 ± 0.02 to 3.21 ± 0.02) did not change significantly with storage temperature and time (Appendix A Table A1). All TBCs were less than 5 log CFU/g, indicating that the HMR products were safe [56]. The heating treatment removed the risk of harmful microorganisms, and freezing maintained the quality of HMR products by preventing the growth of microorganisms. Alizadeh et al. [22] reported that storage temperature influences enzymatic activity in salmon. Low temperature storage in a freezer preserves nutritional composition better than chilled storage. An increase in the activity of microorganisms affects food flavors and produces a foul odor [57]. 

The estimation of shelf life was also evaluated based on the TBCs in HMR products. The TBCs were based on the zero order reaction kinetic. The linear regression of zero order kinetic of TBC is shown in Table 11. HMR products can be stored for up to 236 months at −18 °C, with a target score threshold of 5 log CFU/g. The results of the estimation of shelf life estimation based on sensory properties and TBCs were different. To determine the estimated shelf life, we used the shortest shelf life value between sensory properties (48 months) and TBC (236 months), which changed the HMR product quality. Based on these values, the shelf life of the HMR product containing braised mackerel with radish was 48 months. However, considering the temperature fluctuation during distribution and storage in the market, the shelf life value was multiplied by a safety factor of 0.8 [20]. Therefore, the HMR product containing braised mackerel and radish had a shelf life of up to 40 months.

## 4. Conclusions

The development of the HMR product containing braised mackerel with radish using several food processing technologies, including HFD, SSR, and quick freezing, can maintain the quality of the product. The HFD thawing method resulted in reduced drip loss of materials and a decreased period of thawing, essentially saving production time while maintaining the quality of the raw materials. Using RSM, we determined that the optimal heating conditions for the HMR product to obtain the best sensory properties were 181 °C for 9 min. The texture score of the final products changed significantly owing to the use of different freezing procedures. Quick freezing performed better than slow freezing. Moreover, these treatments resulted in the development of a convenience product with high nutritional value (total protein content of 13.03% with 40% EAA content and total lipid content of 13.42% with 18.07% DHA content). The chemical properties of the products did not change significantly after 90 days of storage. The HMR product containing braised mackerel with radish is safe to consume and has an acceptable quality to consumers for up to 40 months when stored at −18 °C. These findings suggest that braised mackerel with radish is a promising HMR product that follows the recent changes in food preference patterns.

## Figures and Tables

**Table 1:** Composition of sauce used for preparing braised mackerel with radish.

Ingredients	Composition (%)
Red chili paste	22.50
Sugar	12.35
Garlic	12.35
Red chili powder	11.20
Starch syrup	10.00
Sesame oil	6.50
Cooking wine	4.00
Soybean oil	4.00
Brewed soy sauce	1.50
Beef seasoning	1.50
Stir-fried sesame seeds	1.00
Pepper	2.50
Onion powder	4.60
Purified water	6.00

**Table 2:** Independent variables and their levels in the two factors and three points of central composite design for optimizing the heating conditions of braised mackerel with radish.

Independent Variables	Symbol	Coded Level				
		−1.414	−1	0	1	1.414
Temperature	X_1_	166	170	180	190	194
Time	X_2_	7.2	8	10	12	12.8

**Table 3:** Response surface predictive model of overall acceptance and hardness of braised mackerel with radish at different heating temperatures (X_1_) and times (X_2_).

Response	Predictive Model	*R* ^2^	Lack of Fit (*p*-Value)
Overall acceptance	−515.3 + 4.897X_1_ + 17.25X_2_ − 0.013X_1_^2^ − 0.641X_2_^2^ − 0.027X_1_X_2_	98.82%	0.054
Hardness	33223 − 337X_1_ + 15X_2_ + 1.003X_1_^2^ + 27.47X_2_^2^ − 4.06X_1_X_2_	96.93%	0.359

**Table 4:** Predicted and actual values of overall acceptability and hardness of braised mackerel in central composite design.

Std. Order	Heating Condition		Overall Acceptability (score)		Hardness (g)	
	Temperature (°C)	Time (min)	Predicted	Actual	Predicted	Actual
1	170	8	5.03	5.33	1363.99	1271.6
2	190	8	5.86	6.19	1207.46	1142.8
3	170	12	4.48	4.62	859.35	866.67
4	190	12	3.16	3.33	377.75	412.8
5	166	10	6.13	5.91	1161.92	1210.98
6	194	10	5.78	5.52	715.22	724.67
7	180	7.2	4.6	4.24	1424.32	1524.8
8	180	12.8	2.32	2.19	490.27	448.3
9	180	10	8.48	8.52	741.91	741.4
10	180	10	8.48	8.38	741.91	671
11	180	10	8.48	8.57	741.91	811

**Table 5:** Optimal temperature and time for heating braised mackerel with radish using the superheated steam roaster.

	Temperature (°C)	Time (min)
Coded values	0.0625	−0.0269
Actual values	180.625	9.0316
Predicted values		
Overall acceptability	8.49	
Hardness	740.74	
Experimental values (181 °C; 9 min)		
Overall acceptability	8.55 ± 0.10	
Hardness	761.83 ± 78.33	

**Table 6:** Sensory properties of reheated braised mackerel with radish before freezing and after slow and quick freezing.

Sensory Properties	Before Freezing	Quick Freezing	Slow Freezing
Color	8.62 ± 0.11 ^a^	8.52 ± 0.15 ^a^	8.57 ± 0.11 ^a^
Aroma	8.48 ± 0.13 ^a^	8.38 ± 0.13 ^a^	8.33 ± 0.11 ^a^
Flavor	8.52 ± 0.11 ^a^	8.57 ± 0.51 ^a^	8.23 ± 0.10 ^a^
Texture	8.48 ± 0.13 ^a^	8.38 ± 0.11 ^ab^	8.00 ± 0.12 ^b^
Overall acceptance	8.55 ± 0.10 ^a^	8.49 ± 0.07 ^a^	8.29 ± 0.07 ^a^

Note: Scores are expressed as the mean ± standard error of mean (SE. The means of each sensory property with different letters are significantly different according to Tukey’s test (*p* < 0.05).

**Table 7:** Proximate composition of braised mackerel with radish.

Compositions	Unit	Content
Calories	kcal/100 g	208.38
Carbohydrates	g/100 g	8.87
Crude Protein	g/100 g	13.03
Crude fat	g/100 g	13.42
Sugars	g/100 g	4.32
Saturated fat	g/100 g	3.65
Trans fat	g/100 g	0.06
Cholesterol	mg/100 g	27.2
Sodium	mg/100 g	332.5
Moisture	g/100 g	63.21

**Table 8:** Amino acid profile of braised mackerel with radish.

Amino Acids	Content	
	g/100 g	%
Aspartic acid	1.28	9.89
Threonine	0.61	4.71
Serine	0.55	4.25
Glutamic acid	1.98	15.30
Proline	0.54	4.17
Glycine	0.73	5.64
Alanine	0.83	6.41
Valine	0.66	5.10
Isoleucine	0.57	4.40
Leucine	1.05	8.11
Tyrosine	0.35	2.70
Phenylalanine	0.52	4.02
Histidine	0.57	4.40
Lysine	1.19	9.20
Arginine	0.82	6.34
Cysteine	0.17	1.31
Methionine	0.41	3.17
Tryptophan	0.11	0.85
Total	12.94	100

**Table 9:** Fatty acid profile of braised mackerel with radish.

Fatty Acids	Content	
	mg/100 g	%
Lauric acid (12:0)	10	0.09
Tridecanoic acid (13:0)	10	0.09
Myristic acid (14:0)	380	3.27
Pentadecanoic acid (15:0)	80	0.69
Palmitic acid (16:0)	2310	19.88
Heptadecanoic acid (17:0)	90	0.77
Stearic acid (18:0)	670	5.77
Arachidic acid (20:0)	60	0.52
Heneicosanoic acid (21:0)	10	0.09
Behenic acid (22:0)	30	0.26
Tricosanoic acid (23:0)	10	0.09
Lignoceric acid (24:0)	10	0.09
Palmitoleic acid (16:1)	400	3.44
Elaidic acid (18:1)	50	0.43
Oleic acid (18:1)	2980	25.65
Linolelaidic acid (18:2)	10	0.09
Linoleic acid (18:2)	920	7.92
γ-Linolenic acid (18:3)	10	0.09
Linolenic acid (18:3)	150	1.29
*cis*-11-Eicosenoic acid (20:1)	300	2.58
*cis*-11,14-Eicosadienoic acid (20:2)	20	0.17
*cis*-8, 11, 14-Eicosatrienoic acid (20:3)	10	0.09
*cis*-11,14,17-Eicosatrienoic acid (20:3)	20	0.17
Arachidonic acid (20:4)	120	1.03
Erucic acid (22:1)	60	0.52
Eicosapentaenoic acid (20:5)	720	6.2
Nervonic acid (24:1)	80	0.69
Docosahexaenoic acid (22:6)	2100	18.07
Total saturated fatty acid	3670	31.58
Total unsaturated fatty acid	7950	68.42
Total fatty acid	11,620	100

**Table 10:** Sensory properties of braised mackerel with radish during storage for 90 days at three different freezer temperatures.

Temp. (°C)	Day	Color	Aroma	Flavor	Texture	Overall
−13	0	8.43 ± 0.11 ^abc^	8.43 ± 0.11 ^abcd^	8.52 ± 0.11 ^abc^	8.48 ± 0.11 ^a^	8.50 ± 0.02 ^a^
	15	8.43 ± 0.11 ^abc^	8.67 ± 0.11 ^ab^	8.67 ± 0.11 ^a^	8.19 ± 0.09 ^ab^	8.49 ± 0.01 ^a^
	30	8.07 ± 0.05 ^cd^	8.36 ± 0.09 ^abcd^	8.55 ± 0.10 ^ab^	8.05 ± 0.05 ^b^	8.29 ± 0.02 ^bcde^
	45	8.07 ± 0.05 ^cd^	8.48 ± 0.10 ^abcd^	8.21 ± 0.08 ^abcd^	7.98 ± 0.05 ^b^	8.25 ± 0.02 ^def^
	60	8.02 ± 0.02 ^d^	8.05 ± 0.03 ^d^	8.02 ± 0.02 ^d^	8.02 ± 0.02 ^b^	8.13 ± 0.03 ^f^
	75	8.05 ± 0.03 ^cd^	8.07 ± 0.05 ^d^	8.10 ± 0.06 ^bcd^	8.00 ± 0.00 ^b^	8.19 ± 0.02 ^ef^
	90	8.00 ± 0.00 ^d^	8.10 ± 0.07 ^cd^	8.05 ± 0.05 ^cd^	8.05 ± 0.05 ^b^	8.13 ± 0.03 ^f^
−18	0	8.43 ± 0.11 ^abc^	8.43 ± 0.11 ^abcd^	8.52 ± 0.11 ^abc^	8.48 ± 0.11 ^a^	8.50 ± 0.02 ^a^
	15	8.71 ± 0.10 ^a^	8.43 ± 0.11 ^abcd^	8.24 ± 0.10 ^abcd^	8.29 ± 0.10 ^ab^	8.50 ± 0.02 ^a^
	30	8.26 ± 0.09 ^bcd^	8.74 ± 0.09 ^ab^	8.55 ± 0.10 ^ab^	8.12 ± 0.07 ^ab^	8.40 ± 0.04 ^abc^
	45	8.14 ± 0.07 ^cd^	8.50 ± 0.10 ^abcd^	8.50 ± 0.10 ^abcd^	8.10 ± 0.09 ^ab^	8.32 ± 0.06 ^bcde^
	60	8.14 ± 0.08 ^cd^	8.40 ± 0.11 ^abcd^	8.38 ± 0.10 ^abcd^	7.95 ± 0.08 ^b^	8.25 ± 0.05 ^def^
	75	8.10 ± 0.07 ^cd^	8.29 ± 0.10 ^bcd^	8.36 ± 0.10 ^abcd^	8.05 ± 0.05 ^b^	8.26 ± 0.03 ^cdef^
	90	8.00 ± 0.0 ^d^	8.14 ± 0.07 ^cd^	8.24 ± 0.08 ^abcd^	8.02 ± 0.02 ^b^	8.23 ± 0.03 ^def^
−23	0	8.43 ± 0.11 ^abc^	8.43 ± 0.11 ^abcd^	8.52 ± 0.11 ^abc^	8.48 ± 0.11 ^a^	8.50 ± 0.02 ^a^
	15	8.57 ± 0.11 ^ab^	8.48 ± 0.11 ^abcd^	8.43 ± 0.11 ^abcd^	8.48 ± 0.11 ^a^	8.50 ± 0.02 ^a^
	30	8.21 ± 0.07 ^bcd^	8.69 ± 0.09 ^ab^	8.57 ± 0.10 ^ab^	8.17 ± 0.07 ^ab^	8.41 ± 0.02 ^ab^
	45	8.12 ± 0.07 ^cd^	8.79 ± 0.09 ^a^	8.62 ± 0.10 ^a^	8.12 ± 0.07 ^ab^	8.42 ± 0.02 ^ab^
	60	8.29 ± 0.09 ^bcd^	8.74 ± 0.09 ^ab^	8.68 ± 0.09 ^a^	7.95 ± 0.08 ^b^	8.42 ± 0.01 ^ab^
	75	8.10 ± 0.09 ^cd^	8.76 ± 0.08 ^ab^	8.31 ± 0.10 ^abcd^	8.00 ± 0.10 ^b^	8.40 ± 0.01 ^abc^
	90	8.10 ± 0.07 ^cd^	8.57 ± 0.11 ^abc^	8.57 ± 0.10 ^ab^	8.02 ± 0.02 ^b^	8.34 ± 0.01 ^bcd^

Note: Scores are expressed as mean ± standard error of mean (SE). The means of each sensory property with different letters are significantly different according to Tukey’s test in a two-factorial design analysis (*p* < 0.05).

**Table 11:** Linear regression equation for overall acceptance and total bacterial count of braised mackerel with radish at selected order.

Storage (°C)	Overall Acceptance (at First Order)	Total Bacterial Count (at Zero Order)
−13	y = 2.1392 − 0.0006x	y = 3.1282 − 0.0002x
−18	y = 2.1406 − 0.0004x	y = 3.1339 − 0.0003x
−23	y = 2.1396 − 0.0002x	y = 3.1929 − 0.0004x

## Data Availability

Data supporting reported results are available upon request.

## Appendix A

**Table A1:** Total bacterial count (TBC) of braised mackerel with radish during storage for 90 days at three different freezer temperatures.

Days	Temperature		
	−13 °C	−18 °C	−23 °C
0	3.17 ± 0.03 ^a^	3.17 ± 0.03 ^a^	3.17 ± 0.03 ^a^
15	3.17 ± 0.03 ^a^	3.13 ± 0.04 ^a^	3.21 ± 0.02 ^a^
30	3.11 ± 0.02 ^a^	3.17 ± 0.01 ^a^	3.20 ± 0.00 ^a^
45	3.16 ± 0.03 ^a^	3.18 ± 0.00 ^a^	3.16 ± 0.03 ^a^
60	3.17 ± 0.05 ^a^	3.17 ± 0.01 ^a^	3.18 ± 0.00 ^a^
75	3.15 ± 0.02 ^a^	3.12 ± 0.01 ^a^	3.14 ± 0.01 ^a^
90	3.18 ± 0.02 ^a^	3.14 ± 0.01 ^a^	3.17 ± 0.02 ^a^

Note: The data on TBC (log CFU/g) are expressed as the mean ± standard error of the mean (*SE*). The means of TBCs with different letters are significantly different according to Tukey’s test in a two-factorial design analysis (*p* < 0.05).
